# An introduction to retrodictive qualitative modeling as an emerging method on affective variables in SLA research

**DOI:** 10.3389/fpsyg.2023.1081502

**Published:** 2023-02-20

**Authors:** Yulan Gu

**Affiliations:** School of Foreign Languages, Sichuan University of Arts and Science, Dazhou, Sichuan, China

**Keywords:** retrodictive qualitative modeling, L2 affective variables, complex dynamic systems theory, emerging theory, qualitative modeling

## Abstract

Investigating second language acquisition (SLA) *via* a complex dynamic systems theory (CDST) involves much intuition, and operationalizing the dynamic constructs is hard in research terms. In the present study, we contend that the commonly used quantitative data analysis methods such as correlational works or structural equation modeling fail to examine variables as part of a system or network. They are mostly based on linear rather than non-linear associations. Considering the major challenges of dynamic systems research in SLA, we recommend that innovative analytical models such as retrodictive qualitative modeling (RQM) be used more. RQM manages to reverse the usual direction of research by actually beginning from the end. More especially from certain outcomes and then moves backward to find why specific elements of the system led to one outcome rather than the others. The analytical procedures of RQM will be elaborated on and also exemplified in the SLA research, more specifically for investigating language learners’ affective variables. The limited body of research using RQM in the SLA domain is also reviewed followed by some conclusive remarks and suggestions for further research into the variables of interest.

## Introduction

Retrodictive qualitative modeling (henceforth RQM) is a framework for research influenced by the conceptual research of David Byrne, who was a social complexivist. RQM found its way into second language acquisition (SLA) research first by Dörnyei (2014) as he invited researchers to adopt a dynamic perspective in their exploration of the psychological constructs related to language learning see Dörnyei and Ryan (2015). Thus, given this invitation, researchers in the field of SLA felt the need to apply methods which can be compatible with the complex and dynamic systems theory (CDST) (Hiver and Al-Hoorie, 2019). One of these methods was RQM as this research method aims to explore how cognitive, affective, or behavioral patterns that are complex and attractor-governed appear in dynamic, self-organized developmental process. Also, it can be considered a research template based on case studies in the field of SLA whose unit of analysis is a complex system (Byrne, 2013; van Geert and Steenbeek, 2014). In particular, as the unit of analysis in RQM is marked by complexity, in this method, there is an inherent focus on the situated context-dependent nature, and time-scaled variation as well as multicausality, which can provide deeper insights into the dynamic trajectories of affective variables in SLA research. Hence, regarding the application of the method in the current and future SLA studies, RQM can be conceptualized as a method of investigating complex dynamic constructs or behaviors which begins with an emergent cognitive, affective or behavioral construct of interest and goes back into time. Initially, it can sound strange but the underlying belief is that, in the analysis process, we look back at the reality, as the prefix RETRO implies, significant interpretive layers of reality emerge (Byrne, 2010). This looking back at the reality gives more tangible explanations with respect to the real-time experiences of language learners in their classroom ecology see Larsen-Freeman (2016), which is another support for the significance of the method in SLA research.

More specifically, RQM differs from other, dominant, methods of analysis in the field of SLA which take a more variable-centric perspective and look forward in an attempt to predict empirically the kind of emergent patterns or try to make forward-looking suggestions for what may occur (Byrne and Callaghan, 2014). The order of this procedure is reversed in RQM. Therefore, the first step is to identify the attractor states or the outcomes of a given domain like foreign language enjoyment within the affective domain. The next step is to look backward to identify the dynamic procedure that accounted for these. In language studies, for example, a work of research may start with monitoring and determining significant affective constructs of language learning like foreign language enjoyment in an English as a foreign language course. Next, the researcher might want to examine, using the saturated self-report data, how the specific attractor states seen in the educational context emerged.

It is worth noting that although the name of RQM may make all SLA researchers think the method is necessarily qualitative, in practice, it does not have to be. RQM is rather a mixed approach and involves multiple methods of research, not exclusively qualitative in type. RQM does not necessarily involve a formal modeling. Nevertheless, the underlying rationale for RQM is truly univocal, pointing to the fact that it is essential to look backward (what “retrodictive” actually means) from dynamic and complex emergent patterns to find out about the dynamic, context-dependent procedures that contributed to the emergence of those specific states. RQM also emphasizes that this is possible in each stage of research having its basis in the design of former stages. The RQM standard procedure, as described by Dörnyei (2014) with regard to the application of the method in SLA studies, is a sequential array of practical steps beginning with the identification of qualitatively divergent attractor states (“Q”) which a system has reached followed by an inductive evidence collection while going backward (“R”) to obtain a model for the dynamic procedure (“M”) which led to those attractor states (Dörnyei, 2014). RQM aims to delve into the prominent and typical outcomes of an SLA cognitive, affective or behavioral construct, and then to investigate the underlying processes that have multiple causes (which is termed as “signature dynamics” too) and make each of these outcomes and their dynamic trajectories idiosyncratic.

## Complex dynamic system in SLA

By CDST, language learning is seen as a complex system of cooperating elements or factors. In this system, language progress arises in a communicative setting as a consequence of a process of self-organization (Verspoor et al., 2021). This theory calls for a revitalized awareness of intra-individual diversity and non-linear patterns of progress, as well as a holistic-system perspective (Lowie and Verspoor, 2019). Thus, in such a system, analysis of individual trajectories is necessary.

The idea that different aspects of SLA issues should be specifically addressed from a complex dynamic approach was initially put out by Larsen-Freeman (1997). Since that time, CDST has attracted the attention of numerous SLA researchers who have studied various areas of the field, including language acquisition and development (Larsen-Freeman, 2016), language ecology (van Lier, 2008), language variations (Cooper, 1999), and psychology of language learning and teaching (Dörnyei, 2014).

Researchers now have a fresh viewpoint that questions the fundamental assumptions underlying scientific investigation using CDST. In particular, traditional beliefs that a language’s features are conveyed from a proficient speaker to a beginner student or from an intrinsic universal system to a manifest language usage have been challenged in SLA (Larsen-Freeman, 2020). According to the CDST theory, language develops as a result of a learner using it in a situation involving interaction with other language users. CDST takes an ecological perspective. In other words, learners are not cut off from their learning environment or their learning process. This is because complex systems are frequently seen as cross-disciplinary since they are linked, self-organizing, and co-adaptive.

## Advantages of RQM in SLA studies

In the SLA domain, RQM has been more to the interest of investigating psycholinguistic and sociolinguistic matters (Chan et al., 2015; Hiver, 2017). Beyond SLA research, several researchers interested in investigating complexity in different social sciences suggested using the retrodictive model as the best method which adopts a mathematical modeling framework from the higher-order complexity line of research (e.g., Larsen-Freeman and Cameron, 2008; Byrne, 2009, 2011). The best introductory source to RQM in the field of SLA, as suggested by Hiver and Al-Hoorie (2019) is Dörnyei’s (2014). If the core rationale for using the RQM in SLA studies and its constituent elements (procedural steps) are justified, a basic knowledge of how to elicit and analyze longitudinal data can most possibly suffice to make the suggested procedure useful for all researchers.

Retrodiction differs from the prediction in that the latter entails inferring future events or behaviors from the data available now. However, the former is the interpretation of the current data based on past observations. Emergence, in a dynamic system, often prevents thinking about clear-cut future events regardless of the former states of the system as general rules are inconceivable regardless of a particular background (Byrne, 2013). What makes things still more indefinite is the possibility of agency in social sciences (Al-Hoorie, 2015; Larsen-Freeman, 2019). When things are to a great extent indefinite, how can we generate any knowledge that can be applied to the complex underlying causes of outcomes and procedures that can be seen in the surrounding world? How could the logical forward-looking claims be made by saying “if a certain thing is done, what happens next”? These issues should, certainly, be considered in a well-integrated SLA research program (Dörnyei, 2014, as cited in Hiver and Al-Hoorie, 2019).

In an attempt to solve these issues in the application of the method in SLA studies, RQM tries not to only focus on the causes of variation in complex systems. Rather, it delves into the dynamic outcomes and the inherent quality of the emergent effects. After establishing a sound knowledge of outcomes and effects, the next step is, as suggested by Byrne (2011), to look backward at how the systems developed over time or reflect the systems’ gaining or losing stability while changing through time. Thus, the kind of research questions addressed in RQM focus more on a historical, past-looking provision of evidence for present time events. When this evidence is provided, the study can be further developed to the inquiry of the potential prospective evidence (i.e., probabilistic anticipations) in a retrospective manner (Byrne and Callaghan, 2014). Instances of research questions for exploring affective variables in SLA research are provided below as suggested by Hiver and Al-Hoorie (2019).

• What emergent outcomes are conceivable for a human L2 learner’s system of developing a certain affective variable (e.g., anxiety, boredom, enjoyment)? Why these at the cost of others?
• What is the current state of this system, and why is it as it is?
• How has the system of a particular affective factor become what it is at the present time?
• Which combination of similar constructs or cases lead to divergent outcomes in terms of a certain affective factor?
• Which combination of similar constructs or cases lead to identical outcomes in terms of a certain affective factor?
• What changes and adaptations has a system had before it reached the present state of a given affective factor?
• How can older trajectories of an affective variable get organized?
• What conditions are involved in the changes made to the L2 learner’s affective variable? Which factors together can lead to the experience of certain affective variables?
• What causal explanation can be provided to the understanding of how the system reached its current state?
• What type of future projection would the observers count on?

## Procedures of a retrodictive approach

Retrodiction helps SLA researchers to find out how a current state became what it is now. Thus, it involves making inferences about certain conditions by moving backward and excavating the underlying causes (Sayer, 1992). Such a retrodictive procedure has its roots in social sciences and has always looked for revealing the complex and context-dependent causal mechanisms (Downward and Mearman, 2007). Many, if not all complexivists, admit that the search for a cogent and detailed causal explanation for system outcomes is essential for research with the aim of both elucidating observable states and being useful (Byrne and Callaghan, 2014). With the emergence of CDST in the field of SLA, the difficulty pertaining to case studies lies in the generation of knowledge from certain analytical units with the potential of being expanded to more than a particular case (Lowie and Verspoor, 2019). If retrodiction is used as a basic research design, SLA researchers can investigate what dynamic systems are and what they actually do, and probably more importantly, how they turn into their current state. Thus, RQM manages to reflect better the global outcomes and procedures of variation by unraveling how that variation is made.

The first stage of RQM involves discovering the attractor states or the system outcomes which are to be investigated (for instance, an outcome of language learners’ anxiety or boredom), and then sampling the individual learners purposefully. Thus, typical language learners who show a high level of the variable of interest should be selected. Of note is that the number of patterned outcomes is limited corresponding to the dominant attractor states characterizing the system through time in the space of the state (Hiver, 2015a).

The second step involves purposively sampling the typical cases that are adequately representative of the recurrent outcome (e.g., the affective variable of interest such as foreign language enjoyment) and are prototypical representative of a certain pattern. Next, the data elicitation techniques are employed to investigate the target outcome. This would involve a comprehensive account of exemplary behavior, and elaborations of the features of the most prototypical instances. It usually entails the collection of an array of information sources from traditional focal groups or through interviews, self-administered questionnaire surveys and also observations. As, in its most general sense, the RQM is problem-centered, retrodiction needs a set of qualitative and quantitative approaches to research to go beyond the employment of discipline-oriented techniques (Mearman, 2006). It should be noted that the outcomes on their own just reflect part of a whole, which do not necessarily be the most fruitful and revealing part. Therefore, discovering and elaborating on the latent constituent elements of the system is just half of the whole research procedure (Hiver and Al-Hoorie, 2016).

The next step of RQM involves tracing the trajectories of dynamic growth using the time-scaled data so as to reveal the idiosyncratic signature dynamics (i.e., the strongest causal mechanisms underlying a system) and contribute to the conceptualization of how the system led to that outcome (e.g., to analyze the process through which a language learner develops boredom). In the end, when such growing complexities are better scrutinized, the practical or realistic reflection of the given outcome (i.e., what it appears to be and the way it affects other systems) is investigated in an analytical way.

## Review of RQM studies in SLA

The number of studies conducted in the SLA domain using the RQM is limited. Here, the few studies conducted so far with this approach will be reviewed. The procedures and findings can be insightful for a future body of research.

First of all, Dörnyei (2014) drew attention to the rise of CDST in SLA research, yet admitted that it was hard to operationalize this dynamic approach in actual research. Then, he introduced RQM as an effective research method that reversed the typical direction of research by beginning from the end, which means beginning from the outcomes of a system, and then going back to find why specific elements of the system led to the emergence of a particular outcome at the cost of the other conceivable outcomes. More specifically, he illustrated two class-based research projects in which the language class was considered as a dynamic system, and the system outcome alternatives were the different learner archetypes (e.g., motivated, demotivated, and passive) which were found in the L2 class.

As one of the first studies using RQM in the field of SLA, Chan et al. (2014) employed RQM to explore L2 learner motivation in Hong Kong. The researchers started their study by first requesting an instructors’ focused group to identify prominent learner archetypes in the classrooms. Then, based on the instructors’ descriptive reports, the researchers conducted in-depth interviews with the prototype learner in each group. as a result, they were able to learn more about the “signature dynamics” of the motivational system as it relates to the various prototypes. They demonstrated how RQM might be applied practically to identify the underlying causes of a specific conclusion involving language learners, and their retrodictive technique was efficient.

One of the best exemplary studies on the use of RQM was conducted by Hiver (2017). He investigated EFL teachers’ performance, motivation, and well-being. He looked into the performance, motivation, and well-being of Language instructors. Using the RQM, he explored how EFL instructors maintained their professional equilibrium and efficiency in the face of their demanding and anxiety-provoking workplaces. The first piece of study (Hiver, 2015b) provided proof that instructors, in response to conflicts unique to the classroom, developed a higher-order psychological entity known as the “teacher immunity.” According to this exploratory research, Hiver contended that teacher immunity grows into a double-nature protective form. Sometimes, the emergent outcome might act as a useful protection to help teachers to continue to be committed and to succeed. Yet, in other forms it can adversely affect the individual’s performance and turn into an occupational threat. Thus, Hiver decided to explore the dynamic behaviors of the adaptive instructor immunity outcome and the maladaptive instructor immunity outcome.

Particularly, with regard to the procedures of RQM, Hiver (2017) used the RQM to first discover and analyze the notably common outcomes of L2 teacher immunity. Afterward, he used a powerful two-step identification procedure through triangulating exploratory focal group data (from different teachers as well as teacher trainers) with quantitative questionnaire surveys of a bigger sample of EFL professionals to substantiate the validity of the outcome features and categories both theoretically and emergently. Using the triangulated data collection, the researcher discovered accurate teacher immunity prototypes and categorized them in four emergent groups:

(1) adaptively immunized (i.e., those enjoying a strong, advantageous kind of teacher immunity),

(2) maladaptively immunized (i.e., those having a constant, unproductive kind of teacher immunity),

(3) immunocompromised (i.e., those not having any integrated kind of teacher immunity);

(4) moderately immunized (i.e., those with half-way characteristics of teacher immunity).

The researcher discovered these primary archetypes and after that took a further step to scrutinize the network of constituent elements and the primary underlying dynamic patterns of growth–or the system’s signature dynamics–that led to the observed outcomes. These conventional data sources allowed the researcher to trace back the global dynamic procedure on which all four categories were based in a self-disciplined array of four steps–triggering, linking, rearrangement and stabilization. The researcher’s further fine-tuned analysis revealed the complex causal underpinnings that led these systems to generate case-wise trajectories or directions of development for specific outcomes and prototypes. Eventually, the researcher analyzed how the emergent outcomes represented in professionals’ affective variables and attitude, teaching in practice, and undertaking and endurance in their working environments. The researcher indicated that teacher immunity was represented in the incentives, mentalities and performance of different prototypes. Teacher immunity indirectly affected teachers’ range of action and reactions to the situational requirements of their instructions. Moreover, teachers’ self-concept, their perseverance in achieving their goals as well as their self-efficacy were related to the particular outcomes of teacher immunity. Thus, this research concluded that teacher immunity allows teachers to endure challenges, use their motivation resources and remain productive in their routine work conditions.

In a more recent work of research, Elahi Shirvan and Talebzadeh (2020) drew attention to the recent shift in SLA research from negative to positive psychology, and the arising dominance of the dynamic phase in this field of study. These researchers acknowledged that the recent works of research attempted to micro-map the anxiety that foreign language learners experienced as well as the foreign language enjoyment they felt from a dynamic approach. Elahi Shirvan and Talebzadeh (2020) acknowledged that the distinctive dynamics of language learners’ anxiety, which develops as a negative emotion, and enjoyment, which emerges as a positive emotion, had not previously been investigated. Influenced by Dörnyei’s (2014) study, they used RQM as a cutting-edge method to explore at the distinctive dynamics of these two learner-related constructs. *Via* focal-group interviews with a group of educators about their learners’ anxiety and enjoyment in their classes, they were able to pinpoint the enjoyment and anxiety archetypes. They also conducted in-depth interviews with a prototype L2 student from each archetype in order to discover more about the patterns and trajectories that lead to a specific outcome or attractor state, by following and examining the dynamic events in the language class. These findings offered useful insights about the dynamic trends that result in distinctive archetypes of enjoyment and anxiety and also the use of RQM for investigations of the enjoyment and anxiety dynamics.

The most recent work of research enlightened by the RQM seems to be Wang’s (2021) study which explored the second language learning motivation of 5 Chinese undergraduates who learned Bulgarian as their academic field of study in Bulgaria in a 1-year instructional course. This study focused on a scarcely investigated second language learning context, in which the L2 was related to a host population of limited ethno-linguistic vitality. According to the theoretical basis of the second language learning motivational self-system and the ideal multilingual self, and guided by the rules and regulations of RQM, this research discovered 3 different patterns of motivation that emerged within the 1-year experience of studies in a foreign country (i.e., Bulgaria): lowered motivation with a poor ideal Bulgarian self; unstable and changing motivation with a lowered ideal Bulgarian self; and changing motivation with an enhanced ideal Bulgarian self. A comparison of the emergent patterns showed that: (a) L2 learners’ agentic understanding of their layover in the host country had a significant effect on their cultural concerns, thus affecting the their ideal Bulgarian self; (b) the decrease/increase of the ideal Bulgarian self was a main reason for the intensification or impoverishment of the students’ motivation for learning the Bulgarian language; (c) an ideal multilingual self can compensate for the weakened ideal Bulgarian self, through motivating directly the language learners’ learning while the ideal Bulgarian self was lowered.

## Conclusive remarks

As already raised by several researchers especially Chan et al. (2015), there are major methodological issues with doing L2 empirical research inspired by the CDST. Overall, the main concern is that the outcomes of exploring dynamic systems, especially those with human systems (e.g., L2 learners) are hard to predict as it is practically not possible to know beforehand how the different factors work hand in hand with each other (Haggis, 2008). This issue was openly expressed by Larsen-Freeman and Cameron (2008) too. They emphasized that how a complex system behaves is not entirely random, yet it is not fully predictable either. What poses serious problems for researchers is the low predictability and the failure to enlist possibly relevant factors before doing the research. RQM was introduced basically to solve this issue, as it allowed researchers to rely on the self-organizing capacity of a system (i.e., its tendency to raise the regular quality of the initially fleeting, flowing, and complex behavior of the system).

Therefore, RQM uses the adjusting power of self-organization to add predictability to system behavior and make it researchable. As the main word *retrodiction* shows, the conventional way of doing research is reversed in RQM. At first, the end states or archetypes are identified in the L2 learning system’s behavior and then a backward trace is taken to unravel the dynamic trajectories leading to the conditioned states. This is how the reasons why the system could have ended up with a specific outcome is tracked back. The existing literature on RQM applied in SLA research has provided supporting evidence for the effectiveness of the method since the language learning process as well as the language learners’ or teachers’ development of cognitive, affective constructs and behavior are marked by complexity and dynamicity that lend themselves to productive inquiries of causal mechanisms. As the existing literature is still limited in size, many L2 learning constructs remain to be explored further in RQM in the prospective line of research.

## Suggestions for further research

Several L2 teacher- or learner-related constructs have been explored so far using the RQM. Examples are immunity, motivation, well-being, equilibrium, and professional or academic achievements. One line of future investigations can build on the findings of these studies (already done) and explore programs of context-specific interventions for maladaptive teacher or learner outcomes that exerts effects on the self-organized mechanism and assesses ideas, which have partly been suggested somewhere else (Hiver and Dörnyei, 2017), for re-initiating the process at main steps in its self-organized direction and directing it to a more constructive outcome, for example by suggesting a set of revolutionary coping strategies and building resolution narratives. With self-organization, there is a hope of introducing change to every individual in every context (Hiver, 2017). Language learners or teachers faced will low motivation, immunity, equilibrium and the like can be made motivated and self-efficacious again.

Another line of research can set aside the previous literature, and begin to explore unexplored L2 teachers’ or learners’ cognitive, affective or behavioral constructs through the RQM. There are several still under-researched affective variables, for instance, that await being explored in the light of the CDST with an appropriate research methodology. RQM has the capability of exploring the causal mechanisms underlying affective variables such as L2 learning-related boredom, enjoyment, anxiety, and stress among other factors. RQM-led works of research can pave the way for later interventional studies to control or reduce the effect of the causal mechanisms that account for negative emotions or attitudes among language learners.

Finally, as mentioned previously, the use of the method provides both SLA researchers and L2 teachers with deeper awareness about the real-time experiences of L2 learners in the ecology of a second or foreign language classroom see Larsen-Freeman (2016). Thus, it can be applied in future research to explore the signature dynamics of the teacher-student interactions in the L2 classroom as well as both learners’ and teachers’ mindsets about these interactions.

## Author contributions

The author confirms being the sole contributor of this work and has approved it for publication.

## References

[B1] Al-Hoorie A. H. (2015). “Human agency: Does the beach ball have free will?,” in *Motivational dynamics in language learning*, eds Dörnyei Z. MacIntyre P. D. Henry A. (Bristol, UK: Multilingual Matters), 55–72.

[B2] Byrne D. (2009). “Complex realists and configurational approaches to cases: A radical synthesis,” in *The SAGE handbook of case-based methods*, eds Byrne D. Ragin C. C. (Thousand Oaks, CA: SAGE), 101–112.

[B3] Byrne D. (2010). “Comparison, diversity and complexity,” in *Complexity, difference, and identity*, eds Cilliers P. Preiser R. (Dordrecht: Springer), 61–75.

[B4] Byrne D. (2011). *Applying social science.* Bristol: Policy Press.

[B5] Byrne D. (2013). Evaluating complex social interventions in a complex world. *Evaluation* 19 217–228. 10.1177/1356389013495617

[B6] Byrne D. Callaghan G. (2014). *Complexity theory and the social sciences: The state of the art.* New York, NY: Routledge.

[B7] Chan L. Dörnyei Z. Henry A. (2014). “16. Learner Archetypes and Signature Dynamics in the Language Classroom: A Retrodictive Qualitative Modelling Approach to Studying L2 Motivation,” in *Motivational dynamics in language learning*, eds Dörnyei Z. MacIntyre P. Henry A. (Bristol, UK: Multilingual Matters), 238–259. 10.21832/9781783092574-018

[B8] Chan L. Dörnyei Z. Henry A. (2015). “Learner archetypes and signature dynamics in the language classroom: A retrodictive qualitative modelling approach to studying L2 motivation,” in *Motivational dynamics in language learning*, eds Dörnyei Z. MacIntyre P. D. Henry A. (Bristol, UK: Multilingual Matters), 238–259.

[B9] Cooper D. (1999). *Linguistic attractors: the cognitive dynamics of language acquisition and change*. Amsterdam: John Benjamins.

[B10] Dörnyei Z. (2014). Researching complex dynamic systems: ‘Retrodictive qualitative modelling’ in the language classroom. *Lang. Teach.* 47 80–91.

[B11] Dörnyei Z. Ryan S. (2015). *The psychology of the language learner revisited.* London: Routledge.

[B12] Downward P. Mearman A. (2007). Retroduction as mixed-methods triangulation in economic research: Reorienting economics into social science. *Cambridge J. Econ.* 31 77–99. 10.1093/cje/bel009

[B13] Elahi Shirvan M. Talebzadeh N. (2020). Tracing the signature dynamics of foreign language classroom anxiety and foreign language enjoyment: A retrodictive qualitative modeling. *Eurasian J. Appl. Linguist.* 6 23–44.

[B14] Haggis T. (2008). Knowledge must be contextual: Some possible implications of complexity and dynamic systems theories for educational research. *Educ. Philos. Theory* 40 158–176.

[B15] Hiver P. (2015a). “Attractor states,” in *Motivational dynamics in language learning*, eds Dörnyei Z. MacIntyre P. D. Henry A. (Bristol, UK: Multilingual Matters), 20–28.

[B16] Hiver P. (2015b). “Once burned, twice shy: The dynamic development of system immunity in language teachers,” in *Motivational dynamics in language learning*, eds Dörnyei Z. MacIntyre P. D. Henry A. (Bristol, UK: Multilingual Matters), 214–237.

[B17] Hiver P. (2017). Tracing the signature dynamics of language teacher immunity: A retrodictive qualitative modeling study. *Modern Lang. J.* 101 669–699. 10.1111/modl.12433

[B18] Hiver P. Al-Hoorie A. H. (2016). A dynamic ensemble for second language research: Putting complexity theory into practice. *Modern Lang. J.* 100 741–756. 10.1111/modl.12347

[B19] Hiver P. Al-Hoorie A. H. (2019). *Research methods for complexity theory in applied linguistics.* Bristol, UK: Multilingual Matters.

[B20] Hiver P. Dörnyei Z. (2017). Language teacher immunity: A doubleedged sword. *Appl. Ling.* 38, 405–423. 10.1093/applin/amv034

[B21] Larsen-Freeman D. (1997). Chaos/complexity science and second language acquisition. *Appl. Ling.* 18, 141–165. 10.1093/applin/18.2.141

[B22] Larsen-Freeman D. (2016). Classroom-oriented research from a complex systems perspective. *Stud. Second Lang. Learn. Teach.* 6 377–393. 10.14746/ssllt.2016.6.3.2

[B23] Larsen-Freeman D. (2019). On language learner agency: A complex dynamic systems theory perspective. *Modern Lang. J.* 103 61–79.

[B24] Larsen-Freeman D. (2020). “Complex dynamic systems theory,” in *Theories in second language acquisition*, (London: Routledge), 248–270.

[B25] Larsen-Freeman D. Cameron L. (2008). Research methodology on language development from a complex systems perspective. *Modern Lang. J.* 92 200–213. 10.1111/j.1540-4781.2008.00714.x

[B26] Lowie W. Verspoor M. (2019). Individual differences and the ergodicity problem. *Lang. Learn.* 69 184–206. 10.1111/lang.12324

[B27] Mearman A. (2006). Critical realism in economics and open-systems ontology: A critique. *Rev. Soc. Econ.* 64, 47–75. 10.1080/00346760500529955

[B28] Sayer A. (1992). *Method in social science.* London: Routledge.

[B29] van Geert P. Steenbeek H. (2014). The good, the bad and the ugly? The dynamic interplay between educational practice, policy and research. *Complicity* 11 22–39. 10.29173/cmplct22962

[B30] van Lier L. (2008). “The ecology of language learning and sociocultural theory,” in *Encyclopedia of language and education: Vol. 9. Ecology of language*, 2nd Edn, eds Creese A. Martin P. Hornberger N. H. (Boston: Springer Science+Business Media), 53–65.

[B31] Verspoor M. Lowie W. de Bot K. (2021). Variability as normal as apple pie. *Linguist. Vanguard* 7:20200034. 10.1515/lingvan-2020-0034

[B32] Wang L. (2021). The motivational dynamics of learning a foreign language of limited ethnolinguistic vitality during a study abroad. *Int. J. Multiling.* 10.1080/14790718.2021.1960357

